# A theoretically designed scale-up intervention increases adoption of an effective school nutrition program ‘SWAP IT’: outcomes of a randomised trial

**DOI:** 10.1186/s12966-026-01887-z

**Published:** 2026-03-13

**Authors:** Rachel Sutherland, Demi Herdegen, Jannah Jones, Courtney Barnes, Nicole Nathan, Katie Robertson, Anna Rayward, Adrian Bauman, Tameka McFadyen, Andrew Milat, Philip Morgan, Penny Reeves, Christopher Oldmeadow, Serene Yoong, Megan Cobcroft, Simon Chiu, John Wiggers, Luke Wolfenden

**Affiliations:** 1 School of Medicine and Public Health, University of Newcastle, University Drive, Callaghan, NSW 2308 Australia; 2 Hunter New England Population Health, Locked Bag 10, Wallsend, NSW 2287 Australia; 3 Population Health Research Program, Hunter Medical Research Institute, 1/Kookaburra Circuit, New Lambton Heights, NSW 2305 Australia; 4 Sydney School of Public Health, University of Sydney, Camperdown, NSW 2050 Australia; 5 Office of PVC - Health, Medicine and Wellbeing, University of Newcastle, University Drive, Callaghan, NSW 2308 Australia; 6 NSW Ministry of Health, Agency for Clinical Innovation, Reserve Road, St Leonards, NSW 2065 Australia; 7 School of Education, University of Newcastle, University Drive, Callaghan, NSW 2308 Australia; 8 Hunter Medical Research Institute, Kookaburra Cct, New Lambton Heights, NSW 2305 Australia; 9 Faculty of Health/School of Health and Social, Development/Institute for Health Transformation, Deakin University, 221 Burwood Highway, Burwood, Victoria 3125 Australia; 10 New South Wales Ministry of Health, Centre for Population Health, Reserve Road, St Leonards, NSW 2065 Australia

**Keywords:** Scale-up, School, Nutrition, Dissemination, Implementation, Adoption

## Abstract

**Background:**

Scale-up (i.e., the intentional effort to increase the reach and impact of an intervention) of effective school-based interventions is recommended to improve public health nutrition and prevent chronic disease. However, limited evidence to guide effective scale-up exists. This study aimed to assess the effectiveness of a theoretically designed multi-component scale-up intervention, designed using the Theoretical Domains Framework and Behavior Change Wheel, to increase the adoption of an evidence-based school nutrition program (SWAP IT) within primary schools in NSW Australia. We also identify any differences in the characteristics of schools adopting the program and describe the fidelity of delivering, and reach of each scale-up strategy.

**Methods:**

A parallel-group randomised controlled trial was conducted across 11 Local Health Districts (LHD) in New South Wales Australia (*n* = 337 schools). Primary schools which had not previously adopted the SWAP IT school nutrition program and used an existing parent communication app (Audiri) in each LHD were randomised to receive a theoretically designed multi-component scale-up intervention (*n* = 169 schools) or to a waitlist control, exposed to a single discrete scale up strategy (*n* = 168 schools). The scale-up intervention consisted of nine scale-up strategies: three ‘vertical’ strategies aiming to increase LHD Health Promotion Units capacity to scale-up and six ‘horizontal’ strategies aiming to overcome school barriers to adopting the programs. The primary outcome was school adoption of SWAP IT objectively assessed via electronic records at 6-months, assessed via logistic regression adjusting for baseline and stratification variables.

**Results:**

Following the 6-month multi-component scale-up intervention, significantly more schools in the scale-up intervention group (*n* = 67/169, 40%) had adopted the SWAP IT program compared to schools in the control group (*n* = 0/168, 0%) (*p* < 0.001). Schools with a higher enrolment of Aboriginal and Torres Strait Islander students were more likely to adopt the program (*p* = 0.02), however no other school characteristics were associated with program adoption. Most scale-up strategies were delivered by LHD Health Promotion Units to schools with high fidelity (72–100%), however reach of the strategies varied widely (4-100%).

**Conclusions:**

A theoretically designed approach to scale-up, increased schools’ adoption of an evidence-based school nutrition program across a large and socio-economically and geographically diverse population. Program adoption rates were similar across socio-demographic and geographic characteristics. This approach can inform efforts of improving public health nutrition equitably through large scale adoption of school nutrition initiatives.

**Trial registration:**

The trial was prospectively registered on 13/02/2023 with the Australian New Zealand Clinical Trials Registry (ACTRN12623000145606). https://anzctr.org.au/Trial/Registration/TrialReview.aspx?ACTRN=12623000145606.

## Background

Poor dietary intake has been the leading preventable risk factor for illness, death, and disability both in Australia and worldwide for more than two decades [1]. Consistent with international data, in Australia, child dietary intake differs considerably to that recommended by dietary guidelines, increasing children’s risk of future chronic disease [2, 3]. As one of the few settings affording universal access to children [4], and as the school day accounts for up to half of children’s daily energy intake [5], school-based nutrition programs have consistently been recognised as an opportunity to improve child diet intake. Accordingly, delivering effective school-based nutrition interventions at scale has been recommended by leading health agencies internationally including the World Health Organization (WHO) and American Heart Foundation [6, 7]. In Australia, this focus has been on improving the nutritional quality of foods and beverages sold through school canteens [8], with comparatively less attention given to improving child nutrition intake via improving the nutritional quality of school lunchboxes via programs such as SWAP IT, despite approximately 90% of students bringing a packed lunch on any given school day [9].

Despite the public health benefits, effective school-based nutrition interventions are rarely scaled-up [10, 11]. ‘*Scaling-up’ is the deliberate effort to increase the impact of successfully tested health interventions so as to benefit more people”.*^12^ Few attempts to scale-up effective interventions have been reported in the literature, and most of these have failed to successfully implement them with sufficient reach or fidelity to achieve population level improvements in nutrition [13]. The limited use of theory to guide scale-up efforts has been identified as a key factor influencing the success of implementation at scale [14]. Indeed, scaling-up evidence-based health interventions is a considerable challenge with recent reviews identifying a variety of factors including human and financial resources, alignment with strategic priorities and stakeholder willingness and acceptability, operating at multiple levels that may impede effective scale-up [15, 16]. Effective scale-up of nutrition programs must include strategies capable of addressing key barriers such as leadership, staff workload, limited skills, knowledge or resources and community support, to their adoption and integration in schools.

The WHO Expand-Net framework suggests efforts to scale-up must consider characteristics of the interventions that may make them more amenable for adoption and implementation at scale within a school; characteristics of schools (‘host organisation’) where the intervention is being implemented; and the capability of the ‘resource team’ or agency/s responsible for supporting adoption, implementation and scale-up [12, 17]. Specifically, they underscore the importance of identifying ‘scalable’ interventions, that is, those well aligned with the contexts of the organisation that it is to be implemented. Resource teams must also have the capability to enact strategies that address barriers to adoption of the intervention by the host organisation [17]. 

While scale-up frameworks provide helpful conceptual guidance, scale-up is a neglected area of scientific enquiry. Just 2% of research papers in the field of public health focus on scale-up [18]. In particular there is a paucity of research testing the effectiveness of efforts to scale-up health policies or programs [10, 19, 20]. For school nutrition programs specifically, a Cochrane review identified just three trials testing strategies to deliver effective interventions ‘at scale’ – defined in the review as being delivered to more than 50 schools [21]. This leaves agencies responsible for population wide adoption of nutrition programs needing to invest in strategies in the absence of evidence of how best to do so. There are also concerns that efforts to scale-up programs may inadvertently generate health inequities [13]. Intervention generated inequities may occur if an intervention is better suited, more accepted, or appropriate to some population groups over others [22]. In school-based interventions, this may include programs being more readily adopted or implemented in schools serving more socioeconomically advantaged communities, or those with greater organisational capacity and resources. Inequities may also arise, when health policies or programs disproportionately reach or are implemented with greater fidelity in schools located in major cities compared with regional or remote areas, or in schools serving communities experiencing higher levels of socioeconomic disadvantage or enrolling higher proportions of Aboriginal and Torres Strait Islander students, who are the Traditional Owners of the land in Australia and continue to experience persistent health inequities relative to non-Indigenous Australians [16]. This is a particular risk when scaling-up, as variation in context, such as population characteristics and needs, is likely greater. This increases the complexity of ensuring program adoption and delivery is equitable [23]. However the outcomes of scale-up efforts are rarely reported by population subgroups, limiting our understanding of the extent to which this is the case [24]. In particular, scale-up outcomes are infrequently examined by school location, socioeconomic context, or Aboriginal and Torres Strait Islander student enrolment. Understanding how scale-up can occur in a way that helps to addresses and does not further exacerbate inequities is a current priority in the field of implementation science [25, 26]. Addressing equity throughout the scale-up process is also a recognised evidence priority for national prevention agencies in Australia, intended to foster more impactful and equitable prevention of chronic disease [27]. 

This study, therefore, aims to assess the effectiveness of a multi-component scale-up strategy in increasing the adoption of an effective healthy lunchbox program (SWAP IT) within primary schools located in New South Wales, Australia. Secondary aims include examining the characteristics (e.g. socio-economic, geographics, proportion of Aboriginal and/or Torres Strait Islander students) of schools adopting the SWAP IT school nutrition program; and the fidelity of delivering, and reach of, the scale-up strategies.

## Methods

### Ethics and trial registration

The research was conducted and is reported in accordance with the requirements of the Consolidated Standards of Reporting Trials (CONSORT) Statement, the Standards for Reporting Implementation Studies (StaRI) Statement and checklist and TIDIER checklist. Ethics approval was obtained via the following Human Research Ethics Committees: Hunter New England (2019/ETH12353); University of Newcastle (09/07/26/4.04) and New South Wales (NSW) Department of Education (2018247). The trial was registered prospectively with the Australian New Zealand Clinical Trials Registry (ACTRN12623000145606) on the 13/02/2023.

### Aim, study design and setting

**Fig. 1:**
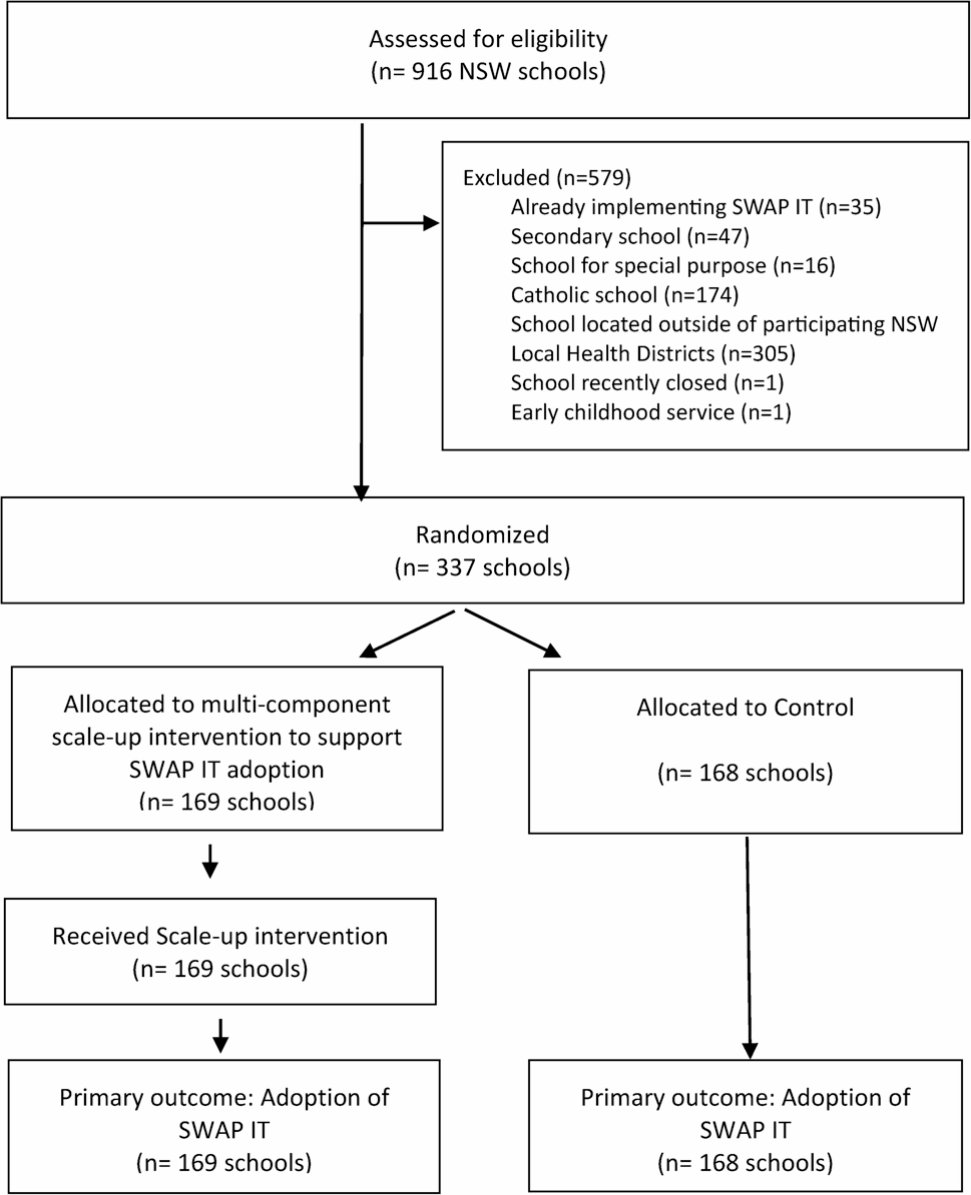
Flow diagram describing schools progress to 6 months

This study assessed the effectiveness of a multi-component scale-up strategy in increasing the adoption of an effective healthy lunchbox program (SWAP IT) in primary schools located in New South Wales, Australia, examined differences in the characteristics of schools adopting SWAP IT and assessed the fidelity of delivering, and reach of the scale-up strategies. A parallel-group randomised controlled trial design was employed to test the effectiveness of a 6-month theory-based multi-component scale-up strategy compared to a discrete strategy, to achieve large scale adoption of the SWAP IT program within NSW primary schools (Fig. 1. Flow of participants through the study). The trial was conducted in 11 New South Wales (NSW) Local Health Districts (LHDs): Murrumbidgee; Hunter New England; Sydney; Western Sydney; South Western Sydney; South Eastern Sydney; Northern Sydney; Southern NSW; Western NSW; Nepean Blue Mountains; and Illawarra Shoalhaven. The 11 partnering LHDs cover > 70% of the NSW population and include major cities, regional centres as well as rural and remote areas [28].

### Sample and participants

All schools that met the following criteria were eligible for the study: (i) primary or combined schools which catered for primary school aged children (5–12 years); (ii) from the Department of Education (DoE) or Independent school sectors; (iii) current users of the Audiri school-parent communication platform (i.e. an existing school-parent communication app commonly used by schools to send information to parents electronically and the platform used to implement the SWAP IT program) at baseline; (iv) located within the boundaries of the 11 partnering LHDs (outlined above) and (v) had not previously implemented the SWAP IT nutrition program. The Australian Curriculum, Assessment and Reporting Authority (ACARA) database of schools [29] and project records from Audiri on current users of the Audiri school-parent communication app served as the sampling frame. Schools enrolling secondary students only, schools catering exclusively for children requiring specialist care (e.g. schools catering for severely disabled or autistic specialist care schools), and schools that had already implemented the lunchbox nutrition program were ineligible.

All eligible schools were recruited to the study and included in the assessment of the primary trial outcomes (described below). Additionally, schools were invited to participate in baseline and follow-up data collection activities to assess secondary trial outcomes. For the recruitment of schools to the baseline and follow up data collection activities, we employed a recruitment strategy developed based on reviews we have undertaken to increase survey participation in this setting [30], An information statement outlining the study was emailed to the school principal together with a personalised link to complete an online survey at baseline and 6-month follow up. Schools that did not complete the survey following the initial invitation received up to three reminder prompts (via email or telephone conducted by a trained research assistant) to encourage completion.

### Randomisation and blinding

Prior to the delivery of the first scale-up strategy, an independent statistician randomised schools, using a computerised random number function, stratified by the school size, geographic and socio-economic location of the school (given its association with implementation of school nutrition programs [31, 32] in a 1:1 (scale-up intervention: control) ratio using random block sizes of between 2 and 6. Due to the nature of the intervention, schools were not blinded to group allocation, however interviewers inviting schools to participate in the principal-completed online or telephone survey at baseline and 6-month follow up were not informed of group allocation nor was the statistician undertaking the randomization and analysis. Further, the primary trial outcome (i.e. adoption of the SWAP IT program) was objectively assessed via electronic records on the SWAP IT program website, eliminating reporting bias.

### An effective, scalable school nutrition program – SWAP IT

**Fig. 2:**
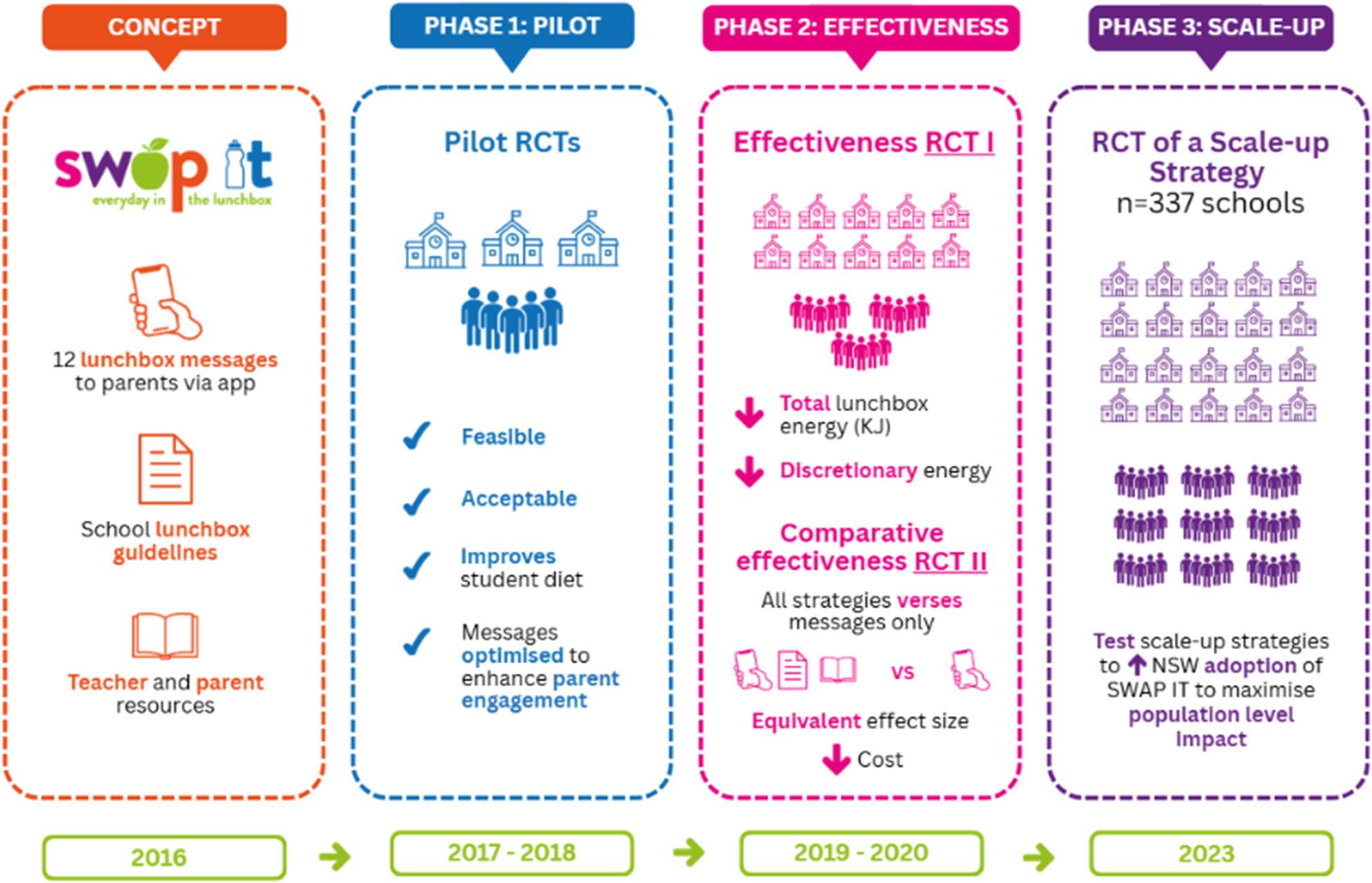
Evolution of SWAP IT

SWAP IT is an effective healthy school lunchbox program (‘SWAP IT’) that aims to improve the contents of children’s lunchboxes by supporting parents/carers to swap what is packed from discretionary (“sometimes”) foods and drinks (those high in fat, salt or sugar) to core (“everyday”) foods and drinks aligned with national dietary guidelines [33]. The program has been extensively evaluated via multiple studies including a randomised controlled trial (pilot) [34], type 1 hybrid implementation effectiveness trial, optimisation study [8] and cost effectiveness evaluation [35] (Fig. 2).

SWAP IT messages are delivered weekly to parents and carers via the school’s usual school-parent communication app (Audiri) for one school term (one message per week), followed by two messages per term on an ongoing basis. The messages provide parents and carers with useful tips and ideas for packing a healthy lunchbox, including recipe ideas and healthy alternatives for ‘sometimes’ foods. Additional resources, including classroom lessons for teachers, school nutrition guidelines and parent’s lunchbox ideas booklets, specifically developed for the SWAP IT program were freely available to schools and parents. All primary (elementary) schools across Australia are able to adopt the program via the program website (www.swapit.net.au). The SWAP IT program was selected as the evidence-based intervention following a comprehensive assessment of its suitability for large scale implementation (‘scalability’). Table 1 provides an a brief overview of the SWAP IT scalability assessment using the Intervention Scalability Assessment Tool undertaken by the research team and stakeholders [36]. 

**Table 1:** SWAP IT scalability assessment using the Intervention Scalability Assessment Tool

ISAT Domain	SWAP IT Assessment
Problem	SWAP IT addresses a prioritised health problem – child diet, a leading risk factor for preventable mortality. [37] Improving child dietary intake is identified within key policy documents internationally and nationally [38, 39] and prioritised by schools and parents. Specifically, SWAP IT addresses primary school aged children’s excess discretionary food intake in school lunchboxes. On any given school day, more than 85% of Australian children bring a packed lunchbox, of which 40% of lunchbox energy is from discretionary foods.[40] Relevant and significant public health issue. [27, 41]
Intervention	SWAP IT is an evidence-based school nutrition program aiming to reduce discretionary food intake via lunchboxes. SWAP IT consists of: (1) short messages designed using behavioural theory to overcome barriers to packing healthy lunchboxes – delivered electronically to parents via existing school-parent communication app (or usual school communication process); (2) resource booklet for families (digital or hard copy) and (3) school resources such as curriculum and school nutrition guidelines templates Program materials and processes are defined and replicable. [42] It is a simple school nutrition intervention, easy for schools to implement, adaptable and low in cost.
Effectiveness	Prior trials have demonstrated significant impacts on child dietary intake, discretionary food reduction, and weight outcomes. [8, 34, 43] SWAP IT reduces discretionary energy packed and consumed in lunchboxes and increase energy from dietary guideline aligned foods. See Fig. 2, outlining phased research demonstrating effectiveness of SWAP IT.
Reach and Acceptability	Program designed to be scalable across diverse primary schools. Previous trials indicate equitable uptake across schools of varying socioeconomic and demographic profiles, including higher uptake in schools with greater Aboriginal enrolment. School principals and parents report high acceptability and feasibility [34]
Implementation Infrastructure	Delivered via existing school communication channels (e.g., school-parent communication app, e.g. Audiri, emails or school newsletters). Minimal burden on teachers. Implementation support systems developed in previous trials.
Cost and Cost-effectiveness	Our previous studies report the cost and cost effectiveness of delivering SWAP IT [35] (AUD$32/student or $0.07/student if delivering electronic messages only [44], though additional cost-effectiveness analyses may be needed at scale. Costs considered low relative to potential public health benefits.
Adaptation	SWAP IT consists of three intervention components: (1) Messages to parents targeting barriers to packing healthy lunchboxes; (2) curriculum materials; (3) parent resources. The intervention is modular and could be adapted for different school contexts or cultural groups while maintaining core elements. A comparative effectiveness trial identified the messages to parents as the CORE intervention component. [44] By adding components, schools can implement a simple parent targeted intervention or add additional elements for a whole of school nutrition intervention.
Sustainability	Intervention effects sustained post trial at 12 months. Potential for ongoing delivery through school communication systems. However, longer-term sustainability beyond research-led support requires further evaluation.
Potential for Scale-up	The characteristics of the SWAP IT intervention (e.g. simple, adaptable, low cost, aligned with existing school systems) together with strategic policy alignment, demonstrated effectiveness and high school and parent acceptability make SWAP IT amendable for scale. Challenges remain in further increasing adoption rates and sustainability at scale.

### Scale-up intervention

The scale-up intervention was co-produced with researchers, stakeholders and consumers using best-practice guidelines including the WHO ExpandNET [12], NSW Guide to Scaling-up Public Health Interventions [17] and implementation science theories and frameworks, including the Theoretical Domains Framework, Behaviour Change Wheel and Theories of Innovation Adoption by Wisdom and colleagues [36, 45–48]. These frameworks were used in a complementary manner: scale-up frameworks (ExpandNET and the NSW Guide) guided decisions about system readiness, governance, resourcing and delivery pathways, while behavioural and implementation theories were applied to understand determinants of school-level adoption and to inform the selection of scale-up strategies.

**Fig. 3:**
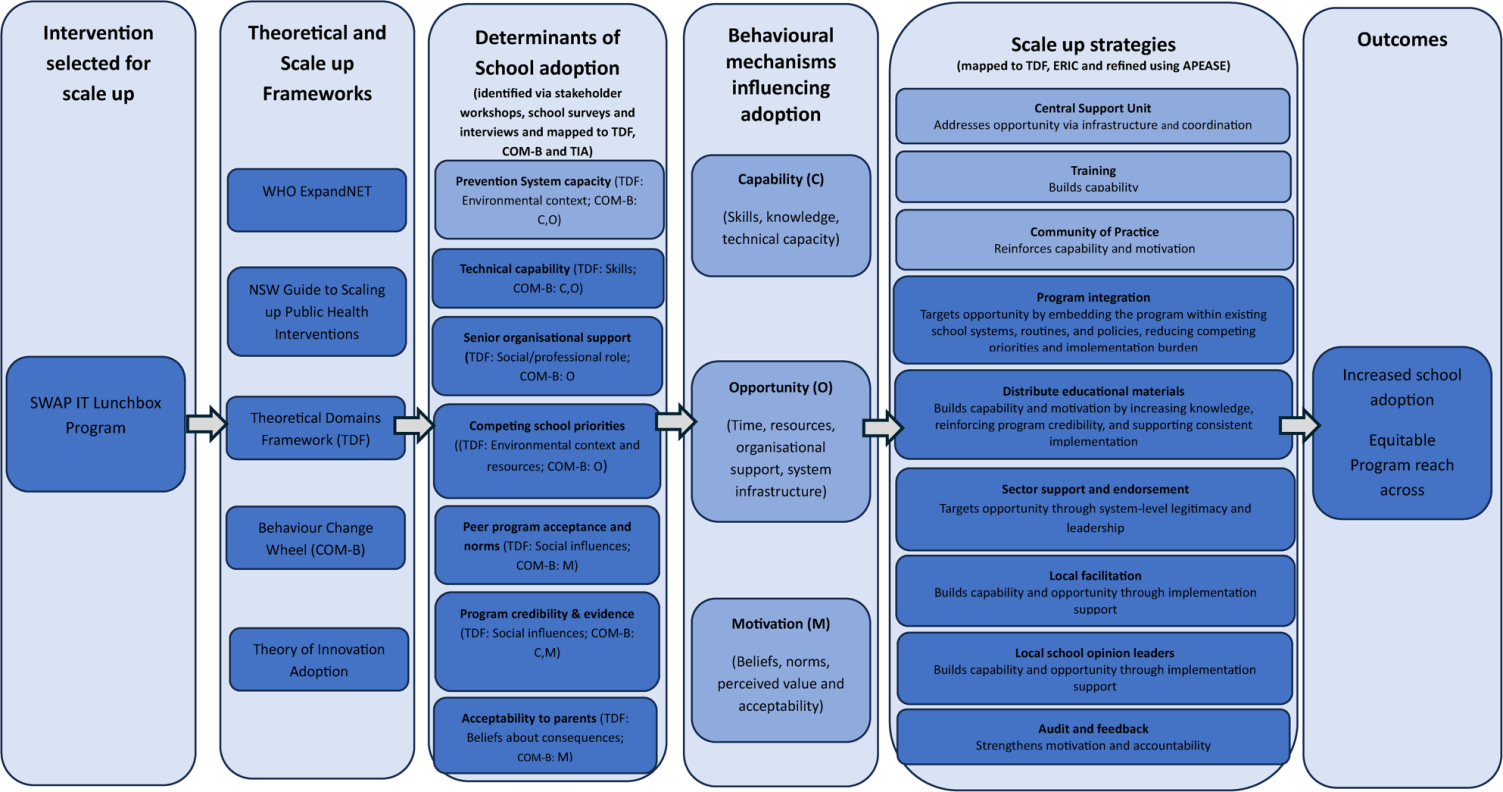
Theory-informed scale-up logic model illustrating how implementation and scale-up frameworks were applied to identify determinants of school adoption, diagnose behavioural mechanisms (COM-B), and select context-adapted scale-up strategies to enhance equitable program adoption

The scale-up intervention was developed following a multi-step process, including: (i) Delivery of a stakeholder workshop where participants (including health promotion practitioners, Health and Education policy makers, Aboriginal Health staff and parents and dietitians) completed a scalability assessment tool to identify SWAP IT as a ‘scalable’ school-based nutrition program (Table 1). This assessment operationalised ExpandNET criteria, including intervention credibility, observability, resource requirements, and alignment with existing systems; (ii) completion of a situation and stakeholder analysis to secure stakeholders assets and infrastructure required for scale-up; and (iii) identification of the barriers and enablers to school adoption of SWAP IT, acquired through key informant interviews and surveys with school principals and staff (Fig. 3). Identified determinants were deductively mapped to relevant TDF domains to provide a theoretically informed behavioural diagnosis of adoption, including domains related to social/professional role and identity (senior organisational support), social influences (peer acceptance and norms), beliefs about consequences (evidence and credibility), environmental context and resources (competing priorities and technical capability), and beliefs about acceptability (parents). Key barriers identified were: senior organisational support, competing priorities, peer acceptance and norms, evidence and credibility, acceptability to parents and technical capability; iv). Pilot scale-up strategies: Using the Behaviour Change Wheel [47], these TDF-mapped determinants were then synthesised into capability, opportunity, and motivational barriers to adoption, and appropriate intervention functions were identified. Scale-up strategies were subsequently selected by mapping these functions to discrete implementation strategies using the Expert Recommendations for Implementing Change (ERIC) taxonomy [45, 49, 50] Strategies were prioritised through a structured co-design process with stakeholders using the APEASE criteria [47] to ensure feasibility, acceptability, and alignment with existing delivery infrastructure and then piloted using existing infrastructure in one local health district in a small sample of schools.

The resulting multi-component scale-up intervention (Table 2) consisted of nine scale-up strategies including three vertical and six horizonal strategies delivered over a 6-month period. The vertical scale-up strategies were designed to build the capacity of the resource team (i.e. LHD health promotion staff) whose role is to support schools in implementing evidence based healthy eating interventions, thereby institutionalising the program through system changes (ExpandNET). These included: (1) Centralised Support; (2) Scale-up training and an (3) Implementation working group. The horizonal scale-up strategies were designed to address the identified determinants of program adoption from the host organisation in order to increase the “spread,” or replicate the program across different geographic sites (schools) and extend to a wider area (ExpandNET) [12]. The following scale-up strategies were included: (1) Program integration; (2) Sector support and endorsement; (3) Local facilitation; (4) Develop and distribute education materials; (5) Audit and feedback and (6) Local opinion leaders. The scale-up strategies are detailed in Table 2. Figure 3 outlines the scale-up logic including the determinants of school adoption, scale-up strategies and expected outcomes.

**Table 2:** Overview of the multi-component scale up intervention including aim, mapping to ERIC taxonomy target and description

Scale up strategy	Aim	ERIC Taxonomy Strategy^	Target/ Who	Type (vertical/ Horizontal)	Description
Scale up strategies targeting Local Health Districts					
Central Support Unit	To support the scale-up and implementation of SWAP IT by: 1.maintaining the program website 2. developing and distributing program resources 3. providing ongoing technical assistance to LHDs	Centralize technical assistance	Local Health Promotors	Vertical	**Scale-up strategy implemented by**: Central Support Unit (Research staff) **Description**: A central support function (research unit) was established to deliver the centralised scale up strategies and increase the capacity of health staff to provide local facilitation via the provision of training and regular scale-up working group meetings. **Dose**: 0.5 Full time equivalent (FTE) throughout scale-up intervention **When**: Throughout the 6-month scale-up intervention
Scale-up training	To increase awareness, knowledge and skills of the SWAP IT program and scale-up strategies	Conduct ongoing training	Local Health Districts (Health Promoters)	Vertical	**Scale-up strategy implemented by**: Central Support Unit (Research staff) **Description**: Training was conducted by the Central Support unit to LHDs to increase knowledge, skills and awareness of SWAP IT, scale-up determinants, strategies, capacity to undertake local facilitation and support school implementation. **Dose**: 1.5 h x 1 occasion **When**: At the beginning of the scale-up intervention (or when new health staff commenced in the role) **Fidelity**: Proportion of LHDs supporting SWAP IT invited to attend online training. **Reach**: Proportion of LHDs that attended training
Scale-up working group	To increase health promotion staff capacity to scale-up via provision of expert advice and local knowledge sharing	Create a learning collaborative (CoP)	Local Health Districts (Health Promotion Key Contact)	Vertical	**Scale-up strategy implemented by**: Central Support Unit **Description**: A scale-up working group (which acted as a community of practice) that included of each LHDs involved in the scale-up intervention was established to share scale-up processes and learnings. The working group was facilitated monthly by the Central Support Unit **Dose**: 1 h x 6 occasions **When**: Monthly throughout the 6-month scale up intervention **Fidelity**: Proportion of LHDs invited to all Community of Practice Meetings over the scale-up period. **Reach**: Proportion of LHDs attending at least 4 of the 6 Scale-up working group meetings over the scale-up period.
Scale up strategies targeting Schools					
Program Integration	To increase the awareness and ease of adopting SWAP IT, through changing the systems of registration	Change physical structure and equipment	School (Executive)	Horizonal	**Scale-up strategy implemented by**: Industry partner (Audiri app company) **Components**: Email (x2), website pop-up (x2), website banner, landing page **Description**: All schools (intervention and control) were alerted to the availability of the SWAP IT program via two emails from the industry partner direct to the school. The email and website pop ups aimed to inform the school that SWAP IT had been integrated directly into the school app and could be automatically delivered to the school community (parents), following principal consent, easing the implementation burden on the school. **Dose**: Twice (including 2 pop ups, 2 emails to schools) throughout the 6-month scale-up intervention **When**: At the beginning of the scale up intervention **Fidelity**: Proportion of schools that had not adopted SWAP IT that were sent (1) 2 emails from Audiri (app provider); (2) pop up promoting SWAP IT integration were active from the Audiri home page for eligible schools; (3) a banner promoting SWAP IT was active on the Audiri website and (4) a SWAP IT information page was available on the Audiri website. **Reach**: Proportion of schools with an Audiri account at the time the strategy was sent, and evidence emails sent from Audiri to active users.
		Change record systems			
Develop and distribute educational materials	To address barriers to adoption of SWAP IT via: 1. creating tension for change (e.g. via outlining parent interest and expectations); 2. communicating attractive program attributes (e.g. relative advantage, simplicity, no-cost); and 3. subsequently emphasising norms and experiences of early adopters (e.g. peer acceptance, social norms).	Develop Educational Materials	School (Executive)	Horizontal	**Scale-up strategy implemented by**: Central Support Unit **Components**: Printed Educational pack, Principal email **Description**: Targeted at principals and other school decision makers, such as the school administration manager and parent committee to address perceived barriers to adoption, the strategy initially aimed to create tension for change; and then communicate the attractive program attributes (e.g. simplicity, no-cost). This communication consisted of a printed information pack containing program promotional material, sample program resources and a letter from the LHD Health Promotion Director to encourage program adoption. Additionally, one marketing email was developed targeting principals and sent via an electronic direct mail system. **Dose**: One printed information pack, one Principal email **When**: Beginning of the 6-month scale up intervention (printed info pack), end of the 6-month scale up intervention (principal email) **Fidelity**: Schools that had not adopted SWAP IT were sent a printed information pack and principal email **Reach**: Mail and email sent out without it being returned to sender due to wrong address/undeliverable or without a bounce back due to wrong email address.
		Distribute Educational Materials			
Local Facilitation	To improve schools’ implementation and intervention effectiveness through improving implementation climate (the extent to which implementation is expected, supported, and rewarded).	Facilitation	School (Executive)	Horizontal	**Scale-up strategy implemented by**: LHD Health Promotion staff Components: Webinar, tailored contacts (x 2) **Description**: Health promotion staff from LHDs have developed strong and trusted local relationships with schools for over a decade and represent credible sources of local nutrition expertise. They hosted a webinar in each LHD and used up to three existing school contacts, telephone and face-to-face visits, to assess interest in the program, address any barriers to adoption, and facilitate goal setting and action planning. **Dose**: Two individual contacts were made by Health staff with schools in their local area. For each contact, up to three phone calls were made to the school in an attempt to directly contact the school’s decision maker (principal or nominated school executive) and encourage program adoption. **When**: Middle of the 6-month scale up intervention **Fidelity**: In schools that had not adopted SWAP IT, LHD Health Promotion Officers made 3 attempts (phone or face-to-face) to contact the school principal or suitable executive member to offer personalised support to overcome barriers to adopting SWAP IT. Email correspondence did not count as a successful contact. **Reach**: (1) Principals (or their delegate) responded to each of the three attempts made by LHD Health Promotion Officers. Email correspondence did not count as a successful contact. (2) Schools attended live webinar.
Audit and Provide Feedback	To provide feedback on current performance (i.e. adoption) and persuade schools to adopt the program through reinforcing social norms	Audit and Provide Feedback	School (Executive)	Horizontal	**Scale-up strategy implemented by**: Central Support Unit and LHD Health Promotors **Components**: updated adoption data **Description**: Data from electronic registration records were used to monitor adoption, provide feedback to schools via the communication strategy and help guide execution and targeting of components. The number of school adoptions of the program was embedded within all strategies targeting schools. For example, letters endorsing the program from NSW Ministry of Health included the number of schools that had adopted the program and health promotion officers conducting local facilitations included the number of school adoption the program within their targeted school contact. **Dose**: The number of school adoptions of the SWAP IT program was included within all scale-up strategies to targeting schools. **When**: Throughout the scale up intervention (incorporated into all communication with schools) **Fidelity**: The Central Support Unit updated the number of schools that had adopted SWAP IT monthly, and reported that information to LHDs, Health Promotion Units to include in school communications. **Reach**: Updated audit and feedback data incorporated by LHDs, Health promotion Units to communicate with schools.
Sector support and endorsement	To increase principals’ motivation to adopt SWAP IT, by providing a credible executive level support and endorsement from a health perspective.	Inform local opinion leaders:	School (Executive)	Horizontal	**Scale-up strategy implemented by**: Central Support Unit **Components**: NSW Health Letter, LHD Letter, Education Sector Letter **Description**: Letters from (1) the NSW Ministry of Health (state health department); (2) Education Sector* and (3) NSW Local Health District Health Promotion Directors were delivered to outline the evidence from a health perspective to schools to encourage adoption. The letter from NSW Ministry of Health aimed to endorse the SWAP IT program and provide credible evidence of the impact of the program on health outcomes. The letter from the NSW Local Health District Health Promotion Director aimed to enhance knowledge of the evidence from a local perspective and encourage school adoption of the program by linking program evidence with local health priorities. **Dose**: One letter from each department **When**: Delivered towards the end of the 6-month scale-up intervention **Fidelity**: All schools in the scale-up group that had not adopted the SWAP IT were sent (via mail) a letter from (1) NSW Health and (2) NSW Health Promotion Unit, endorsing the program and encouraging adoption **Reach**: Letters sent out via mail without it being returned to sender due to wrong address/undeliverable.
	To increase the priority of the innovation for principals by having education sector endorse the program which places the program within organisation policy context*	Mandate change			
Engage local school opinion leaders	To engage with school-level influences or opinion leaders that may have a role (or influence) over decision making, including the school administration manager (SAM) and parents via the P&C.	Inform Local Opinion Leaders	School administrators/ parent body	Horizontal	**Scale-up strategy implemented by**: Central Support Unit **Components**: P&C packs **Description**: Given parent acceptability was a key determinant of program adoption, the Parent and Citizen (P&C) or equivalent committees at each school were the target of the strategy. Printed materials that aimed to raise awareness of SWAP IT, provide sample parental materials and influence the parental committees via promoting the number of schools adopting the program (audit and feedback). A call to action was included encouraging P&C committees to request principals to adopt the program. **Dose**: Once **When**: In the final month of the end of the 6-month scale-up intervention **Fidelity**: For schools that had not adopted SWAP IT, the President of the schools’ P&C Committee was sent the printed information pack. **Reach**: Mail sent out without it being returned to sender due to wrong address/ undeliverable.

*Note: Sector support and endorsement from the Education sector was unable to be obtained within the timeframe of the research

^ *ERIC* Expert Recommendations for Implementing Change (ERIC)

### Control group

The SWAP IT program was freely available to all schools across Australia to adopt via registration on the SWAP IT program website (www.swapit.net.au). Schools in the control group were actively exposed to one discrete scale-up strategy—program integration (Table 2). Control schools were sent two behaviourally designed emails that aimed to provide schools with technical capability and ease of program adoption. The SWAP IT program also appeared on the Audiri user dashboard of control schools—whereby schools in the control group were alerted to the SWAP IT program when they logged in to the Audiri platform and invited to adopt if of interest. The information about the SWAP IT program was available on the Audiri user dashboard throughout the duration of the scale-up intervention. No other prompts, alerts or actions by Audiri to encourage adoption were undertaken. Similarly, the control schools were not actively exposed to any other scale-up strategy. The control group were offered the intervention following the final data collection period.

### Study outcomes and data collection

Baseline data were collected March-April 2023 and at 6-months from November-December 2023 following completion of the scale-up period. Adoption data was collected from the SWAP IT program website. School characteristics data were collected from the ACARA database [29].

#### Primary outcome: program adoption

The primary trial outcome was the number and proportion of schools in each group adopting the SWAP IT program at 6 month follow-up. Adoption was defined as the number of schools which registered and executed the lunchbox nutrition program (SWAP IT) (objective measurement), assessed among schools allocated to the intervention and control groups via electronic registration records captured automatically following school registration to SWAP IT. As part of the registration process, schools provided consent for the de-identified registration data to be used for research and evaluation purposes. This outcome was assessed at baseline and approximately 6 months after baseline data collection.

### Secondary outcomes

#### Characteristics of schools adopting the SWAP IT program

Data regarding geographic location (major city, inner regional, outer regional, remote and very remote, using the 2021–2026 Australian Statistical Geography Standard [51], socioeconomic level (most disadvantaged, least disadvantaged), using Australian postal area index of relative socioeconomic advantage and disadvantage 2021 (Socio-Economic Indexes for Areas - SEIFA) [52], school size (total enrolments (large, small school – above and below total enrolments median), Indigenous enrolments (number of schools with > 10% students who identify as Aboriginal and/or Torres Strait Islander), and students who speak a language other than English (LOTE) (number of schools) was assessed via publicly available data using the 2023 ACARA database [29]. 

#### Intervention fidelity and reach of the scale-up strategies

Project records kept by the Central Support Unit (research team), Local Health District Health Promotion Units and the technology app provider were used to determine the proportion of schools that were provided the scale-up strategy as intended (fidelity) and the proportion of schools that received (reach) each of the scale-up strategies (Table 2). The definition of fidelity and reach is outlined in Table 2.

### Sample size

A sample of 175 schools per group was estimated to enable detection of a 10% difference in adoption between groups (intervention 18%, control 8%) with 80% power at a two sided significance level of 0.05 – considered the minimally important difference from a public health perspective that would still expose thousands of additional students to the benefits of the evidence-based program.

### Data analysis

All analyses were conducted using SAS, version 9.4 (SAS Institute) [53]. Analyses of trial outcomes were undertaken under an intention to treat framework. Descriptive statistics were used to describe school characteristics and adoption of the nutrition program. For assessment of school level program adoption, the primary trial outcome, between group differences, were assessed using logistic regression. The model included a term for treatment group (intervention vs. control). There was no missing primary outcome data at follow-up, as program adoption is recorded automatically for all participating schools. All statistical tests were 2-tailed with an alpha of 0.05. Logistic regression models were used to examine associations between school adoption of SWAP IT and each school characteristic including school size (large/small), socio-economic status (most disadvantaged/ least disadvantaged), geographic location (inner regional/ major cities/ outer regional/ remote/very remote), with simple linear regression used for Aboriginal and Torres Strait Islander enrolments (continuous) and language background other than English (continuous). School characteristics were summarised for intervention and control schools [29]. Projects records were used to record fidelity of the scale-up strategy execution and receipt of the strategy. Descriptive statistics were used to summarise the fidelity and reach of the scale-up strategies over 0–6 month scale-up phase.

## Results

The characteristics of schools included in the scale-up sample are provided in Table 3. Characteristics between the intervention and control group were broadly similar with slighter more Government schools, schools classified as least advantaged and inner regional allocated in the intervention group.

**Table 3:** Sample characteristics

School characteristics	Intervention	Control
Number schools	169	168
School enrolments, mean (SD)	337.11 (255.30)	366.13 (301.25)
Sector, *n* (%)		
Government	158 (93)	151 (90)
Non-Government	11 (7)	17 (10)
Socio-economic level		
Most disadvantaged	78 (46)	80 (48)
Least disadvantaged	91 (54)	88 (52)
Geographic location, *n* (%)		
Major Cities	112 (66)	117 (70)
Inner regional	41 (24)	35 (21)
Outer regional	10 (6)	14 (8)
Remote/very remote	6 (4)	2 (1)
Proportion of students that identify as Aboriginal and/or Torres Strait Islander students, mean (SD)	9.54 (12.06)	7.70 (10.67)
Proportion of students who speak a language other than English (LOTE), mean (SD)	32.57 (30.79)	35.0 (31.72)

### Primary outcome: adoption of SWAP IT

At 6-month follow-up, more schools in the intervention group (*n* = 67, 40%) had adopted the program compared to schools in the control group (*n* = 0, 0%) (*p* < 0.01) (Table 4). Schools allocated to the intervention group had significantly higher odds of adopting the SWAP IT program compared with control schools (OR = 152.02, 95% CI: 34.89-∞, *p* < 0.001).

**Table 4:** School adoption of SWAP IT following a 6-month multi-component scale-up intervention by group allocation

	Scale-up intervention group Baseline (*n*-169)	Scale-up intervention group 6-month follow-up (*n*-169)	Control group Baseline (*n* = 168)	Control group 6-month follow-up (*n* = 168)	*P* value
Adoption	0 (0%)	67 (40%)	0 (0%)	0 (0%)	< 0.01

### Secondary outcomes

#### Characteristics of schools adopting the SWAP IT program

Table 5 outlines the associations between program adoption and school characteristics. The only characteristic significantly associated with program adoption was schools with a higher mean number of Aboriginal and/or Torres Strait Islander students enrolled. Schools with a higher mean number of Aboriginal students were more likely to adopt the SWAP IT program (OR 1.03; 95%; CI 1.01–1.06; *p* = 0.02).

**Table 5:** School adoption of SWAP IT following a 6-month multi-component scale-up intervention by school characteristics

	Adoption by school characteristics for scale-up intervention group					
School Characteristic			Adopted, *n* (%)	Not adopted, *n* (%)	Logistic Regression OR (95% CI)	*P* value
School geographic location		**Major cities**	43 (38.39)	69 (61.61)	0.62 [0.12–3.23]	0.79
		**Inner regional**	18 (43.90)	23 (56.10)		
					0.78 [0.14–4.35]	
		**Outer regional**	3 (30.00)	7 (70.00)		
					OR: 0.43 [0.05–3.48]	
		**Remote/very remote**	3 (50.00)	3 (50.00)		
School socio-economic level		**Most disadvantaged**	36 (46.15)	42 (53.85)	1.66 [0.89–3.09]	0.11
		**Least disadvantaged**	31 (34.07)	60 (65.93)		
School size (median)		**Large (> 264 students)**	35 (36.46)	61 (63.54)	0.74 [0.39–1.37]	0.33
		**Small (≤ 264 students)**	32 (43.84)	41 (56.16)		
**School Characteristic**			**Adopted Mean (SD)**	**Not adopted Mean (SD)**	**Simple linear Regression OR (95% CI)**	***P*** ** value**
Aboriginal student enrolments		**Mean percentage of Aboriginal student enrolments**	12.40 (14.49)	7.69 (10.39)	1.03 [CI 1.01–1.06]	*p* = 0.02

### Fidelity and reach of the scale-up strategies

Across all schools, fidelity (scale-up strategies delivered to schools by the Central Support Unit or LHD Health Promotion Units) ranged from 72% (local facilitation) to 100% (program integration, sector support and endorsement, develop and distribute educational materials, local opinion leaders). Reach (defined as scale-up strategy received by schools) ranged from 4% to 100%, with local facilitation consistently less than 50% reach. (Table 6). One scale-up strategy (Sector Support and Endorsement - Education Sector Letter), did not occur as Education Sector endorsement was not received throughout the scale-up period.

**Table 6:** Overview of the multi component scale-up strategies targeting school barriers to scale-up, key components and the fidelity (provided) and reach (received) of each strategy

Scale-up strategy	Components	Fidelity^a^ (%)	Reach^b^ (%)
Program integration	Email 1	100	91
	Email 2	100	89
	Pop Up^c^ 1	100	91
	Pop Up^c^ 2	100	89
	Banner^d^	100	91
	Landing Page^e^	100	91
Sector support and endorsement	NSW Health Letter	100	97
	LHD HPD^f^ Letter (info pack)	100	100
	Education Sector Letter	0	0
Local facilitation	Tailored Contact 1	72	35
	Tailored Contact 2	80	39
	Webinar	99	4
Distribute educational materials	Printed Information Packs	100	100
	Principal Email	100	80
Local opinion leaders	P&C Pack^g^	100	97
Audit and Feedback	Data updated over the intervention period to reflect school adoption and embedded within scale-up strategies targeting schools	100	100

Notes: (a) Fidelity = proportion of schools delivered scale-up strategy as intended; (b) Reach = proportion of schools receiving the scale-up strategy as intended; (c) Pop-ups =Automated message appearing when a webpage is accessed to alert schools to the program ….;d) Banner = a running message appearing at the top of a webpage to alert schools to the program; e) Landing page = the home page of a website; f) LHD HPD = Local Health District Health Promotion Director; g) P&C Pack = Parents & Citizens Pack

## Discussion

Population wide and equitable adoption is an elusive goal of scaling-up evidence-based policies, practices and programs. In a relatively short period of time (6-months), 40% of intervention schools adopted the SWAP IT program, while none of the control schools initiated adoption. Furthermore, our analysis revealed adoption was equitable across a range of socio-demographic and geographic characteristics of schools. Given prior research found SWAP IT yielded improvements in child weight status [43], a reduction in overall and discretionary lunchbox energy both packed and consumed by students, the findings suggests that the scale-up intervention has the potential to support population-level improvements in public health nutrition across many groups [8]. However, as the analysis focused on school-level adoption, further research is required to examine whether benefits are equitably distributed across students within schools.

Both the size of the effect of the scale-up strategy in supporting adoption and the speed in which it occurred were greater than anticipated. For example, within a similar context, the Live Life Well @ School program in NSW, Australia achieved 40% adoption, taking two years to do so, while a school physical activity intervention resulted in 18% uptake within two-years [54]. Systematic reviews have collated outcomes reported across a range of scale-up studies, however reports about the duration and rate of adoption across evaluation periods appears difficult to attain, potentially as many scale-up evaluations occur retrospectively [55]. The speed of intervention adoption is likely influenced by intervention compatibility, relative advantage, its complexity, and cost [56] -intervention features outlined in Rogers’ diffusion of innovation theory – many of which were also appraised as part of the program scalability assessment [57]. Similarly, user-centred design and stakeholder engagement to co-produce innovations may result in the development of innovations that are faster to implement and adopt [56]. The findings suggests that the careful selection of an intervention to scale-up; capable resource teams, and the careful selection of strategies to address school barriers to adoption (as suggested by scale-up frameworks), can yield large improvements in adoption quickly [58]. 

Given one of the key objectives of scaling up evidence-based health innovations is to increase reach, a key concern and widely debated pitfall of scale-up is the potential to exacerbate health inequities [13]. There can be tension between increasing reach and ensuring those most in need of the evidence-based health innovation can access it, particularly when resources are constrained. Our scale-up intervention resulted in equitable adoption of SWAP IT between schools with varying characteristics, with schools including higher proportions of some vulnerable students adopting at a higher rate. Comparison with previous research is difficult, as reporting scale-up outcomes by population subgroups appears rare [59]. For example, a recent review of scale-up, reported there were no studies that assessed the association between adoption and equity [60]. Further, a comprehensive review of reviews of scaling up health and social science sectors published from inception up until 2020, reported just one of 137 included reviews had a focus on equity [60]. We recommend that a core outcome for scale-up studies should include the reporting of outcomes by population subgroups to ensure equity is addressed both in the design and evaluation phases.

### Implications for policy and practice

Overall, the findings of our scale-up intervention differ from other school-based initiatives (e.g.school-based vegetable and fruit breaks), where differences have been observed in implementation. This may be due to the broad acceptability of the SWAP IT intervention, and the limited demands it places on schools to implement it, given its integration within existing digital infrastructure. Future efforts to scale-up and implement other chronic disease prevention programs that take a similar approach are worth exploring. The significant and equitable adoption of SWAP IT within six months suggests that structured, theory-informed strategies, where scale-up strategies have been selected to overcome determinants using the behaviour change wheel and theoretical domains framework, can accelerate translation of evidence into practice, overcoming barriers that often hinder scale-up efforts. For policymakers and practitioners, these findings highlight the importance of embedding implementation science approaches in planning, delivering, and monitoring scale-up initiatives to maximise both reach and equitable health outcomes. Furthermore, the study underscores the need for routine reporting of adoption outcomes by population subgroups to identify and address potential inequities, ensuring health innovations benefit all segments of the community.

### Strengths and limitations

The trial contributes prospective evidence on scale-up processes and outcomes, addressing a significant gap in the literature. Key strengths include the use of objectives measure of adoption; a rigorous study design (RCT), implementation science frameworks to guide scale-up strategy selection, and the reporting of SWAP IT adoption by school subgroups to determine if adoption was equitable. However, a number of limitations are worth considering when interpreting the research findings. While the scale-up occurred across the state of NSW, it represents a single jurisdiction, which may limit generalisability to other contexts. Additionally, while adoption rates were assessed over a six-month period, longer-term follow-up is required to determine the sustainability of adoption over time, particularly once scale-up support and external motivation are reduced. Longer follow-up is also needed to assess whether adoption continues to increase, as schools often require additional time to secure leadership endorsement, align programs with planning cycles, and obtain necessary approvals to support implementation within existing school timelines. Future research could also examine how these scale-up strategies perform under varying policy, resource, and system conditions, including opportunistic or government-led scale-up contexts.

## Conclusions

A theoretically designed approach to scale-up, using scale-up frameworks and implementation science principles to purposefully increased implementer capacity and address known school barriers resulted in a significant increase in schools’ adoption of an evidence-based school nutrition program. The approach produced effective, efficient and equitable outcomes. This provides evidence to policy makers, practitioners and researchers with one model of equitably scaling-up school-based health programs. Whilst a significant proportion of schools adopted the program in the relatively short scale-up window, efforts to further optimise scale-up particularly to improve scale-up strategy fidelity and reach is warranted to deliver population level health outcomes.

## Data Availability

The participant information statement informs participants about the planned or possible future use of information/data. All hard copy information is being stored at the workplace of the research team at Hunter New England Population Health’s secure Wallsend location in locked filing cabinets and secure computer files. Only research personnel and approved staff working with the data will have access to the data. Any electronic data will only be accessible via password protected accounts and any file-sharing will be restricted to members of the project team. All identifying information will be kept for the required 7 years until it needs to be destroyed. Any use of data that is not covered by the current ethics approval will require additional ethics approval before the data is made available. Anyone seeking to access the data will need to contact the lead investigator, along with seeking appropriate ethical clearances. Only once those approvals are granted will de-identified data be shared via an encrypted communication channel.

## Abbreviations

ACARA: Australian Curriculum, Assessment and Reporting Authority
ATSI: Aboriginal and/or Torres Strait Islander
BCW: Behaviour Change Wheel
CONSORT: Consolidated Standards of Reporting Trials
CSU: Central Support Unit
DoE: Department of Education
LHD: Local Health District
LOTE: Language other than English
NSW: New South Wales
RCT: Randomized Controlled Trial
SEIFA: Index of relative socioeconomic advantage and disadvantage
SES: Socio-economic status
StaRI: Standards for Reporting Implementation Studies Statement
SWAP IT: Swap What’s packed in the Box School Nutrition program
WHO: World Health Organization
